# Exercise Ameliorates Immunosenescence: From Mechanisms to Interventions

**DOI:** 10.3390/biology15010058

**Published:** 2025-12-28

**Authors:** Haili Xiao, Jianchang Ren

**Affiliations:** 1 Institute of Sport and Health, Lingnan Normal University, Zhanjiang 524037, China; xiaohl@lingnan.edu.cn; 2 Guangdong Provincial Kay Laboratory of Development and Education for Special Needs Child, Lingnan Normal University, Zhanjiang 524037, China

**Keywords:** exercise, immunosenescence, aging, metabolism, adaptive immunity, innate immunity, health interventions

## Abstract

Aging is an inevitable biological process that affects the function of various organs, including the immune system. Immunosenescence is characterized by diminished immune cell function, weakened immune responses, and imbalances in immune components, which together reduce the body’s resistance to pathogens and increase the risk of age-related diseases in older adults. These conditions pose direct threats to health and significantly impact quality of life and longevity. Therefore, effective strategies to delay aging and maintain immune function are critical areas of research. Emerging evidence suggests that moderate physical activity can significantly enhance immune system functionality, serving as an effective intervention for mitigating immunosenescence. Exercise is known to remodel the metabolism of carbohydrates, fats, and proteins, strengthen communication between organs and the immune system, regulate the functions of both innate and adaptive immune cells, and decrease age-related chronic inflammation. Furthermore, exercise aids in restoring the functionality of aging immune cells through mechanisms such as autophagy activation, mitochondrial optimization, and epigenetic reprogramming. Importantly, a dose–response relationship exists between various exercise modalities and the attenuation of immunosenescence in older adults, with the cumulative amount of lifelong physical activity profoundly influencing the pace of immunosenescence.

## Simple Summary

Aging is a natural process that affects many parts of our bodies, including the immune system, which helps protect us from diseases. As we age, our immune system gradually loses its strength. This results in weaker defenses against infections and a higher risk of illnesses related to aging, such as autoimmune diseases, cancer, heart disease, and Alzheimer’s disease. These health issues can affect our quality of life and how long we live. Researchers are exploring ways to slow down aging and maintain a strong immune system. Recent studies show that regular physical activity can significantly improve how well our immune system works. Exercise helps our bodies process nutrients better, encourages better communication between our organs and the immune system, and reduces inflammation that can come with aging. Additionally, being physically active can help aging immune cells function better by activating certain cell processes. It is important to note that different types and amounts of exercise can have varying effects on immune health. Overall, staying physically active throughout life can greatly enhance our immune system and help us age healthier.

## 1. Overview of Immunosenescence and Exercise as a Modifiable Factor

Immunosenescence refers to the progressive decline in immune system function associated with aging, an inevitable biological process characterized by diminished responsiveness to pathogens, reduced vaccine efficacy, and significantly increased risk of age-related diseases. At the tissue and organ levels, typical hallmarks of immunosenescence include thymic involution, chronic low-grade inflammation (“inflammaging”), and the pathological accumulation of senescent cells (SnCs) [1]. At the cellular and molecular levels, the underlying mechanisms are more complex and interconnected: immune cell subsets undergo substantial alterations, including depletion of naïve CD8+ T cells, excessive accumulation of memory CD8+ T cells, reduced numbers of dendritic cells, and impaired antigen-presenting capacity, collectively compromising the host’s ability to respond to novel antigens [2,3]; senescent cells exhibit characteristics of proliferative arrest and increased intracellular granules while secreting abundant pro-inflammatory factors—the senescence-associated secretory phenotype (SASP)—severely disrupting tissue microenvironmental homeostasis [4]. These pathological changes are driven by multiple upstream mechanisms, including genomic instability, mitochondrial dysfunction, telomere shortening, aberrant epigenetic modifications, and dysregulation of nutrient-sensing pathways (such as NF-κB, mTOR, and JAK-STAT), ultimately leading to systemic, chronic low-grade inflammation and accelerated degenerative changes in tissues and organs [4].

Immunosenescence represents not merely the functional deterioration of the immune system but is a core driving factor for multiple age-related diseases. This process propels the transition from health to pathological states through immune cell imbalance, loss of regulatory capacity, and dysregulation of key signaling pathways [2]. Specifically, immunosenescence significantly increases susceptibility to neurodegenerative diseases (such as Alzheimer’s disease and Parkinson’s disease) and autoimmune diseases in the elderly population [3,5] while also serving as a critical factor contributing to the elevated cancer incidence in older adults [6,7]. Furthermore, declining immune function leads to the attenuated efficacy of cancer immunotherapy, markedly increased incidence and mortality of infectious diseases (such as influenza and pneumonia) [8,9], and elevated cardiovascular disease risk [10]. More critically, these conditions frequently manifest as multimorbidities, with mutual interactions and reciprocal exacerbation, potentially culminating in organ failure and death, thereby severely threatening the quality of life and life expectancy of the elderly. Consequently, developing safe, effective, and readily implementable intervention strategies to delay or reverse immunosenescence has emerged as an urgent priority in geriatric medicine [2].

Among numerous potential interventions, exercise has garnered considerable attention as a non-pharmacological, modifiable lifestyle intervention characterized by multi-system, multi-target regulatory effects. Accumulating evidence demonstrates that regular physical activity not only extends lifespan [11] but also significantly reduces the incidence of metabolic syndrome, cardiovascular diseases, and cancer [12]. As a non-specific yet highly effective intervention strategy, exercise can directly enhance immune cell activity and reshape immune subset distribution at the cellular level [13] while simultaneously delaying the aging process at the systemic level by improving overall metabolic status, modulating the neuroendocrine–immune network [14], and activating anti-inflammatory mechanisms [15].

Growing evidence supports the beneficial effects of exercise on immunosenescence. At the systemic level, long-term, structured exercise programs effectively reduce inflammatory biomarkers (C-reactive protein, interleukin-6, and tumor necrosis factor-α), thereby alleviating the chronic inflammatory milieu in the immune organs of older adults [16,17]. At the cellular level, acute exercise downregulates Toll-like receptor 4 (TLR4) expression on monocyte surfaces, reduces macrophage infiltration, and promotes macrophage polarization from the pro-inflammatory M1 phenotype toward the anti-inflammatory M2 phenotype [18], a process critical for maintaining immune system homeostasis during aging. Systematic reviews further demonstrate that repetitive exercise can suppress the proliferation of senescent and exhausted CD8+ T cells [19], while moderate-to-vigorous aerobic activity stimulates the mobilization and circulation of immune leukocytes (including natural killer cells and T cells) [15,20], thereby enhancing immune surveillance and metabolic health. These mechanisms synergistically enable physical activity to effectively rejuvenate the aging immune system [21,22]. Collectively, current evidence indicates that regular engagement in diverse physical activities—including moderate-intensity aerobic exercise, resistance training, and flexibility exercises—contributes to enhanced immune system function, attenuated immunosenescence, and improved overall physical capacity in older adults [22,23,24].

Although the ameliorative effects of exercise on immunosenescence are widely recognized, the specific pathways and underlying molecular mechanisms require systematic elucidation. Particularly, understanding how exercise orchestrates synergistic actions across multiple dimensions—including metabolism, epigenetics, and inter-organ crosstalk—to reverse or delay immune system aging is of paramount importance for guiding the development of precision exercise prescriptions. Accordingly, this review aims to comprehensively summarize the effects of exercise on immunosenescence, spanning from mechanisms to intervention strategies. Specifically, this review systematically addresses five key aspects: (1) metabolic remodeling—elucidating how the exercise-induced reprogramming of glucose, lipid, and amino acid metabolism improves immune function; (2) organ–immune crosstalk networks—exploring the roles of the muscle–immune axis and gut microbiota–immune axis in the exercise-mediated amelioration of immunosenescence; (3) cellular remodeling—analyzing the differential effects of exercise on innate and adaptive immune cells; (4) molecular mechanisms—revealing key pathways, including autophagy activation, mitochondrial optimization, and epigenetic reprogramming; and (5) intervention strategies—summarizing the differential impacts of exercise type, intensity, and duration on immunosenescence to provide evidence-based guidance for clinical practice and personalized exercise prescriptions.

## 2. Cellular and Molecular Alterations in the Aging Immune System

Immunosenescence is not merely a decline in immune cell numbers but a complex remodeling process characterized by the accumulation of senescent cells, dysregulated signaling pathways, and a chronic low-grade inflammatory state known as “inflammaging” [25,26]. This systemic alteration compromises pathogen clearance and tumor surveillance while paradoxically increasing the risk of autoimmunity [27].

### 2.1. T-Cell Exhaustion and Thymic Involution

The hallmark of adaptive immunosenescence is the progressive involution of the thymus, leading to a restricted output of naïve T cells after the age of 25 [28,29]. Recent evidence suggests that this decline is exacerbated by metabolic reprogramming, where aged T cells exhibit mitochondrial dysfunction and defective glycolysis [30,31]. While the pool of naïve CD8+ T cells shrinks, there is a compensatory expansion of terminally differentiated memory T cells (TEMRA) and oligoclonal populations driven by chronic viral stimulation (e.g., CMV) [32]. Accompanying this process, regulatory T cells (Tregs) increase in number while interleukin-2 (IL-2) production decreases, and this imbalance further impairs the function of effector T cells [28,29]. Unlike functional memory cells, these senescent T cells often express exhaustion markers such as PD-1, TIM-3, and LAG-3 (elevated PD-1 reduces immune response sensitivity and increases tumor risk) and exhibit a senescence-associated secretory phenotype (SASP), releasing pro-inflammatory cytokines that perpetuate tissue damage [4,31]. Targeting these checkpoints with PD-1 or PD-L1 inhibitors may reactivate the cytotoxic function of senescent T cells [33,34]. Furthermore, the TCR repertoire undergoes significant contraction in diversity due to impaired V(D)J recombination efficiency, limiting the host’s ability to recognize novel pathogens [26]. Notably, although γδ T cells constitute a small proportion of the T-cell pool, their reduction in the elderly significantly compromises early tumor surveillance [35].

### 2.2. B-Cell Dysregulation and Age-Associated B Cells (ABCs)

Humoral immunity undergoes distinct remodeling. While total B-cell numbers may remain stable, there is a critical reduction in naïve B cells (associated with the reduced expression of the surface molecule CD23) and a shift towards a pro-inflammatory subset known as age-associated B cells (ABCs, or CD21-low/CD11c+ B cells) [36]. Recent studies highlight that these ABCs are transcriptionally distinct, driven by T-bet expression, and are prone to producing autoantibodies rather than protective neutralizing antibodies [36,37]. Functionally, aged B cells show intrinsic defects in class-switch recombination (CSR) and somatic hypermutation (SHM), explaining the poor vaccine efficacy observed in the elderly population [38]. Based on this, the ex vivo expansion of peripheral blood B cells followed by reinfusion of “rejuvenated” B cells has emerged as a novel therapeutic strategy [39]. The accumulation of these dysfunctional B cells contributes to the inflammatory milieu through the secretion of TNF-α and IL-6.

### 2.3. Remodeling of Innate Immunity and Antigen Presentation

**Figure 1:**
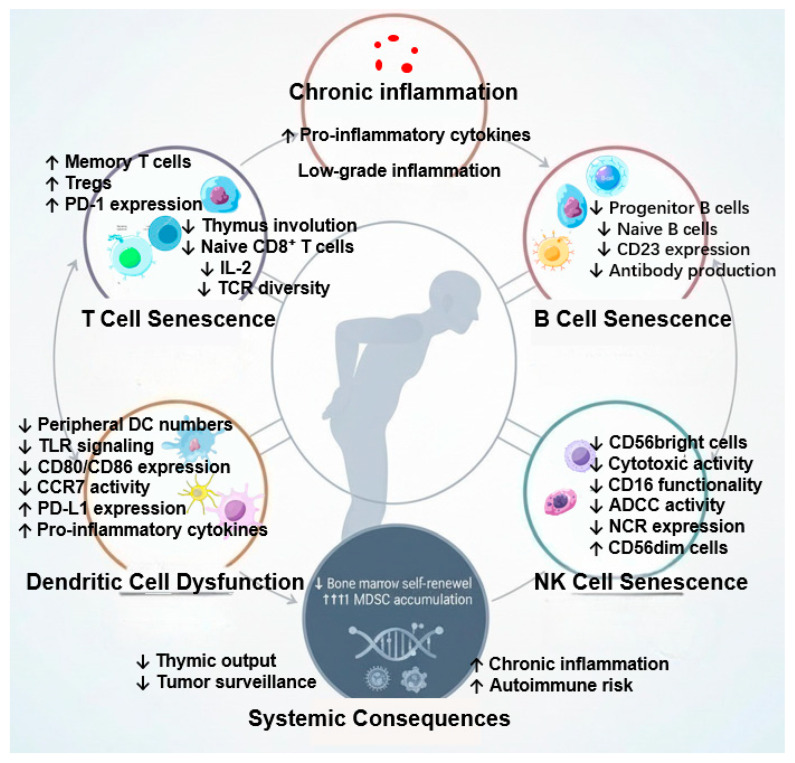
Cellular and molecular alterations in immunosenescence. This diagram illustrates the multidimensional decline in both innate and adaptive immunity in the elderly. Progressive thymic involution leads to a significant decrease in naive CD8+ T cells and a substantial accumulation of memory T cells, alongside increased Tregs and decreased IL-2 production, accompanied by declining TCR diversity and elevated PD-1 expression. Concurrently, the B-cell compartment exhibits declines in progenitor and naive B cells (associated with reduced CD23 expression) with increased effector B cells, resulting in impaired immunoglobulin gene rearrangement and significantly lowered antibody production. Regarding innate immunity, dendritic cells (DCs) show decreased peripheral numbers with reduced antigen uptake, TLR signaling, and CCR7 activity while downregulating costimulatory molecules (CD80/CD86) and upregulating PD-L1 and pro-inflammatory cytokine secretion (e.g., IL-6, TNF-α). Furthermore, natural killer (NK) cells display reduced CD56bright and increased CD56dim subsets, with markedly lower cytotoxic activity due to impaired CD16 function (affecting ADCC) and decreased NCR expression. These cellular alterations, coupled with reduced bone marrow self-renewal, significant MDSC accumulation, and elevated pro-inflammatory cytokines driving chronic inflammation, collectively result in reduced infection clearance, compromised tumor surveillance, and increased autoimmune risk. (Note: arrows indicate the direction of change compared to young individuals: ↑ indicates increase/upregulation; ↓ indicates decrease/downregulation/impairment).

Innate immune cells, the first line of defense, exhibit a “trained immunity” defect with aging. Dendritic cells (DCs) in the elderly show quantitative and functional decline, characterized by impaired micropinocytosis, reduced migration to lymph nodes (linked to diminished CCR7 activity), and defective antigen cross-presentation to T cells [2,25]. At the molecular level, this is linked to altered TLR signaling, the decreased expression of costimulatory molecules (CD80/CD86), and the increased expression of PD-L1 [40]. Natural killer (NK) cells undergo a redistribution from the immature CD56bright subset to the mature CD56dim subset [25]. However, despite their mature phenotype, aged NK cells exhibit reduced per-cell cytotoxicity and impaired cytokine production (e.g., IFN-γ), partly due to signal transduction defects [2]. This reduced activity specifically stems from the altered expression of CD16 (leading to impaired Antibody-Dependent Cellular Cytotoxicity, ADCC) and the decreased expression of natural cytotoxicity receptors (NCRs) [41]. Additionally, the accumulation of myeloid-derived suppressor cells (MDSCs) in lymphoid tissues creates an immunosuppressive microenvironment that inhibits T-cell proliferation, further facilitating tumor immune evasion [25] (Figure 1).

## 3. Mechanisms of Exercise in Ameliorating Immunosenescence

### 3.1. Exercise-Induced Metabolic Remodeling

The immune and metabolic systems share complex regulatory networks, and aging disrupts this homeostasis, creating a vicious cycle of metabolic dysregulation and immune dysfunction known as “immunometabolic exhaustion” [42]. As a central hub connecting nutrition, metabolism, and immunity, the mTOR pathway senses energy status to regulate immune cell differentiation. However, aging-associated mTOR hyperactivation drives insulin resistance and lipid abnormalities while paradoxically impairing T-cell development and pathogen responses [1]. This dysregulation manifests as “inflammaging”, where adipose tissue—acting as a dysfunctional endocrine organ—accumulates senescent cells. These cells release pro-inflammatory factors via the senescence-associated secretory phenotype (SASP), such as IL-6 and TNF-α, which exacerbate systemic insulin resistance [19].

Crucially, regular exercise does not merely consume energy but functions as a “systemic signaling event” that restores immunometabolic flexibility. By optimizing nutrient-sensing pathways (e.g., AMPK/mTOR) and generating bioactive metabolites (e.g., lactate, succinate), exercise breaks the feedback loop of metabolic stress–cellular senescence–inflammatory amplification [43]. This remodeling creates a permissive microenvironment that supports immune surveillance while restraining chronic inflammation.

#### 3.1.1. Glucose Metabolic Reprogramming

Aging is frequently characterized by glucose intolerance and hyperglycemia, which accelerate immunosenescence through multiple mechanisms: suppressing neutrophil chemotaxis and ROS generation [44], promoting pro-inflammatory M1 macrophage polarization [45], and generating advanced glycation end products (AGEs). AGEs bind to their receptor (RAGE) on immune cells, triggering a self-perpetuating “AGEs-RAGE-inflammation” loop that sustains chronic cytokine release (TNF-α, IL-6) [46,47]. Regular exercise dismantles this inflammatory machinery by fundamentally remodeling glucose handling and downstream signaling through three interconnected mechanisms.

Restoration of insulin sensitivity and glycolytic homeostasis. By enhancing skeletal muscle GLUT4 expression and glucose uptake, exercise reduces glycemic fluctuations and limits the formation of AGEs [48,49]. This metabolic stabilization restores the phagocytic and bactericidal capacity of neutrophils and macrophages, which are otherwise compromised by glucotoxicity [50,51].

Lactate as an epigenetic and signaling modulator. Beyond its role as a fuel source, exercise-derived lactate acts as a potent “lactokine” that orchestrates immune plasticity. While acute high-intensity exercise elevates lactate to 20–30 mmol/L [52], producing transient immunosuppression via pH reduction and GPR81 signaling to limit tissue damage [53,54], physiological adaptations to regular exercise are more profound. Recent evidence highlights that lactate serves as a substrate for histone lactylation (e.g., H3K18la) [55]. This epigenetic modification directly activates M2-associated gene expression, driving macrophages from a pro-aging M1 phenotype toward a reparative M2 phenotype [56,57]. Furthermore, lactate supports the oxidative phosphorylation (OXPHOS) of regulatory T cells (Tregs) and memory T cells through MCT1/LDHB, subsets critical for maintaining immune tolerance in the elderly [56,58,59].

Recalibration of the AMPK-mTOR-HIF-1α energy-sensing axis. Exercise counteracts immunosenescence by restoring the balance of cellular energy sensors that are dysregulated in aging. Exercise-induced ATP depletion activates AMPK [60], which acts as a “metabolic brake” on aging by directly inhibiting mTORC1—a complex often overactive in aging that drives pro-inflammatory glycolysis—thereby shifting immune cells towards anti-inflammatory phenotypes (e.g., Tregs, M2 macrophages) [61,62,63]. While acute hypoxia activates HIF-1α to support effector T-cell glycolysis (e.g., Th17) [64,65,66], chronic regular exercise reduces basal HIF-1α overactivation in adipose tissue macrophages, preventing excessive pro-inflammatory polarization [67,68]. These pathways form an interactive network: AMPK inhibits mTOR and modulates HIF-1α, while HIF-1α feedback inhibits mTOR via REDD1 [69,70,71]. By fine-tuning this axis, exercise orchestrates a shift from “Warburg-like” pro-inflammatory glycolysis to mitochondrial oxidative phosphorylation, a metabolic state essential for the longevity and function of memory T cells and quiescent stem cells in the aging host [42,43].

Collectively, these three mechanisms exemplify exercise as a “systems-level metabolic reprogramming intervention”. Unlike pharmacological interventions that target single pathways (e.g., metformin activating only AMPK), exercise simultaneously optimizes substrate availability (glucose), generates bioactive signaling metabolites (lactate), and recalibrates the energy-sensing machinery (AMPK/mTOR/HIF-1α). This multi-pronged approach is particularly critical in aging, where isolated interventions often fail due to redundant pro-inflammatory pathways. For instance, restoring insulin sensitivity alone cannot fully reverse immunosenescence if basal HIF-1α remains elevated; conversely, inhibiting mTOR without addressing hyperglycemia may compromise nutrient-sensing adaptability. Exercise, by contrast, creates a “virtuous cycle”: improved glucose handling reduces the metabolic stress that drives mTOR overactivation → suppressed mTOR enhances AMPK activity → activated AMPK promotes lactate utilization and M2 polarization → M2 macrophages further improve insulin sensitivity in adipose tissue. This self-reinforcing loop underscores why exercise remains the most effective non-pharmacological strategy for delaying immunosenescence.

#### 3.1.2. Lipid Metabolic Optimization

Aging-associated lipid metabolism dysregulation extends far beyond simple fat accumulation, manifesting as a systemic disruption of the adipose tissue–immune cell–circulating lipid network [2,72]. Rather than viewing these components in isolation, emerging evidence positions adipose tissue as a central immunometabolic hub whose dysfunction during aging initiates cascading failures across three interconnected layers. At the tissue level, visceral adipose expansion and hypoxia create a pro-inflammatory microenvironment dominated by M1 macrophages and senescent cells. At the endocrine level, altered secretion of adipokines (adiponectin ↓, leptin ↑) shifts systemic immune tone toward chronic activation. At the metabolic level, dyslipidemia—characterized by elevated pro-inflammatory saturated fatty acids (SFAs) and triglycerides (TGs) coupled with reduced functional high-density lipoprotein (HDL)—directly impairs immune cell membrane integrity, signaling, and survival. Crucially, exercise does not merely “burn fat” but functions as a systemic reprogramming intervention that simultaneously reduces adipose inflammation, restores adipokine balance, and optimizes the circulating lipid profile. This multi-tiered approach breaks the vicious cycle of “adipose dysfunction → immune activation → metabolic deterioration → accelerated immunosenescence,” creating instead a virtuous loop where improved adipose health reinforces immune resilience [72,73].

Adipose tissue remodeling and bidirectional immune–adipocyte crosstalk. The aging process is frequently accompanied by exacerbated visceral fat accumulation and adipose tissue dysfunction, characterized by adipocyte hypertrophy, hypoxia, increased cell death, and the massive infiltration and M1 polarization of immune cells, particularly macrophages [74]. However, this is not a one-way street: activated adipose tissue macrophages (ATMs) and dysfunctional adipocytes engage in bidirectional crosstalk that amplifies local and systemic inflammation. ATMs secrete pro-inflammatory cytokines (TNF-α, IL-6, MCP-1) that further impair adipocyte insulin signaling and lipolysis, while hypertrophic adipocytes release free fatty acids (FFAs) and damage-associated molecular patterns (DAMPs) that activate macrophage NLRP3 inflammasomes [75,76]. This crosstalk forms a local inflammatory microenvironment that spills into circulation, triggering systemic, chronic low-grade inflammation that accelerates immunosenescence. Recent single-cell RNA sequencing analyses reveal that aged visceral adipose tissue harbors at least 13 distinct macrophage subpopulations, with age-associated macrophages (AAMs) expressing CD38 and exhibiting heightened sensitivity to lipopolysaccharide (LPS) alongside reduced efferocytosis capacity—a phenotype that perpetuates the accumulation of dead adipocytes and inflammatory debris [72].

Long-term regular exercise, especially endurance training combined with resistance training, effectively reduces body fat, particularly visceral fat [77], decreases the total macrophage infiltration in adipose tissue, and promotes M1-to-M2 phenotype switching [78]. For example, 8 weeks of voluntary wheel running significantly reduced the proportion of F4/80+CD11c+ M1 macrophages in the epididymal adipose tissue of obese mice, increased the F4/80+CD206+ M2 proportion, and downregulated TNF-α and IL-6 mRNA expression [79]. Subcutaneous adipose tissue biopsies from obese or overweight individuals after exercise training showed the decreased expression of M1-associated genes (CD68, CD11c) and the increased expression of M2-associated genes (CD163, IL-10) [80]. Additionally, exercise alleviates adipose tissue hypoxia, thereby suppressing inflammation initiation [81]. Studies have demonstrated that moderate-intensity exercise, such as Tai Chi, effectively reduces serum inflammatory markers (IL-6, CRP) in obese middle-aged and elderly individuals, improving inflammation associated with adipose tissue dysfunction [82].

Adipokine-mediated endocrine reprogramming and immune modulation. Exercise counteracts immunosenescence by significantly altering the circulating levels and tissue expression of key adipokines. Adiponectin, the most representative anti-inflammatory and insulin-sensitizing adipokine [83], is typically reduced in elderly and obese individuals, whereas regular exercise, particularly long-term endurance training, can elevate its circulating concentrations [84]. Adiponectin activates AMPK and PPARα/γ pathways in macrophages via receptors AdipoR1 and AdipoR2, promoting M2 polarization, suppressing endotoxin-induced TNF-α and IL-6 production, and enhancing phagocytosis and IL-10 secretion [85] while simultaneously inhibiting T-cell proliferation and activation and promoting Treg function, thereby alleviating immunosenescence [86]. Conversely, leptin, a pro-inflammatory cytokine whose levels positively correlate with body fat mass, promotes monocyte/macrophage activation and cytokine production, induces Th1 immune responses, and inhibits Treg development and function [87]. Hyperleptinemia and leptin resistance are common in obesity-associated aging states, and regular exercise, particularly when accompanied by weight loss, can reduce circulating leptin levels [88]. For instance, 12 weeks of aerobic exercise significantly decreased serum leptin in overweight/obese adults, accompanied by reductions in inflammatory markers such as CRP [89]. Importantly, the immunomodulatory capacity of adipokines is influenced not only by their circulating concentrations but also by age-related changes in post-translational modifications and receptor expression on immune cells [2,72].

Circulating lipid profile optimization and membrane-level immune cell protection. Exercise directly ameliorates aging-associated immune dysregulation by optimizing the circulating lipid profile. Dyslipidemia (elevated free fatty acids [FFAs], elevated triglycerides [TGs], and reduced high-density lipoprotein [HDL]) progressively worsens during aging and is closely associated with immune functional decline and chronic inflammation [90,91]. Regarding FFAs, different types exhibit markedly distinct immunological effects. Saturated fatty acids (SFAs, such as palmitic acid) directly activate Toll-like receptor 4 (TLR4) signaling to trigger NF-κB-mediated pro-inflammatory cytokine production [72,92]. Monounsaturated fatty acids (MUFAs, such as oleic acid) exert weaker inflammatory effects than SFAs and may reduce membrane-dependent pro-inflammatory signaling or exert limited anti-inflammatory effects by partially replacing membrane SFAs, though these effects are less pronounced than those of polyunsaturated fatty acids (PUFAs) [93]. PUFAs are categorized into n-6 (arachidonic acid is a precursor of pro-inflammatory eicosanoids, though some metabolites also possess anti-inflammatory activity) and n-3 (renowned for anti-inflammatory and pro-resolution properties via specialized pro-resolving mediators [SPMs]), which often exhibit antagonistic relationships [2]. Although acute exercise transiently elevates FFAs for energy supply, chronic regular exercise significantly enhances the tissue FFA utilization capacity, reducing fasting and postprandial FFAs, especially pro-inflammatory SFAs [94], thereby decreasing aged immune cell exposure to high pro-inflammatory FFA risk. Moreover, reducing basal FFAs through improved insulin sensitivity is critical for alleviating aging-associated chronic inflammation—studies confirm that regular exercise reduces serum FFAs in obese populations, accompanied by decreased CRP and IL-6 [95].

Regarding HDL, its multiple immune protective functions (neutralizing endotoxin to prevent TLR4 activation; inhibiting monocyte adhesion to and migration across the endothelium; directly suppressing monocyte/macrophage inflammatory responses, reducing TNF-α and promoting IL-10 release; preventing macrophage conversion to pro-inflammatory “foam cells” by promoting cholesterol efflux via ABCA1/ABCG1 [96]) are often attenuated during aging due to decreased HDL levels and impaired function. Aerobic and endurance exercise effectively elevates the levels of HDL [97] and not only increases its concentration but also improves its functional quality (e.g., enhancing the cholesterol efflux capacity [CEC] and antioxidant capacity, such as paraoxonase 1 [PON1] activity) [98], providing important support for delaying immunosenescence. Regarding TGs, hypertriglyceridemia commonly associated with aging induces lipotoxicity through uptake of TG-rich lipoproteins (very-low-density lipoprotein [VLDL] and its remnants) by innate immune cells, accompanied by oxidative stress and endothelial dysfunction, exacerbating systemic low-grade inflammation and creating an adverse microenvironment that accelerates immunosenescence. Regular aerobic exercise effectively reduces circulating TGs [68] by increasing skeletal muscle fatty acid uptake and oxidation, reducing hepatic VLDL-TG synthesis and secretion, and improving insulin sensitivity, directly alleviating the pro-inflammatory stimulation and lipotoxic stress of lipid overload on aged immune cells [99].

Collectively, these three layers exemplify adipose tissue as a “systemic immunometabolic command center” whose exercise-induced remodeling exerts pleiotropic anti-aging effects. Unlike targeted pharmacological interventions (e.g., PPARγ agonists that improve adipose insulin sensitivity but may worsen fluid retention; fibrates that lower TGs but do not restore HDL function), exercise simultaneously (1) reduces adipose tissue hypoxia and the M1 macrophage burden, (2) restores the adiponectin/leptin ratio to favor anti-inflammatory signaling, and (3) optimizes the FFA/TG/HDL profile to protect immune cell membranes and receptor function. This creates a self-reinforcing virtuous cycle: reduced visceral fat → ↓ leptin and ↑ adiponectin → M2 polarization in adipose tissue → ↓ systemic IL-6/TNF-α → improved insulin sensitivity → further reduction in lipotoxic FFAs → preserved T-cell and macrophage function [73,100]. Notably, emerging single-cell transcriptomics data reveal that aging-associated changes in adipose tissue are heterogeneous, with distinct macrophage subpopulations responding differently to metabolic challenges. These findings underscore that optimizing lipid metabolism through exercise is not merely a metabolic intervention but a cornerstone strategy for preserving immunological homeostasis and extending healthspan.

#### 3.1.3. Amino Acid Metabolic Regulation

The aging process is frequently accompanied by amino acid metabolic imbalance, which disrupts immune cell function through multiple interconnected mechanisms. Beyond their classical roles as protein building blocks, specific amino acids—glutamine (Gln), arginine (Arg), and branched-chain amino acids (BCAAs: leucine, isoleucine, valine)—function as critical signaling molecules that directly influence immune cell fate and metabolic programming [101]. At the metabolic substrate level, amino acids serve as preferential fuels and biosynthetic precursors for rapidly proliferating immune cells. At the signaling level, amino acids act as mTOR pathway sensors that regulate effector versus regulatory immune cell differentiation. At the microenvironmental level, amino acid availability shapes local immunosuppressive or pro-inflammatory niches. Exercise induces systemic and local changes in amino acid metabolism, and fluctuations in the concentrations of key amino acids and alterations in metabolic pathways directly regulate immune cell function, playing a crucial role in maintaining immune cell functionality during aging [102,103].

Glutamine metabolic remodeling: balancing fuel supply and redox homeostasis. As the most abundant free amino acid in blood [104], Gln serves as a preferentially utilized key fuel (via glutaminolysis for energy supply) and biosynthetic precursor (providing nitrogen/carbon for nucleotide synthesis) for rapidly proliferating or highly activated immune cells, such as lymphocytes and macrophages [105]. Additionally, Gln is an essential substrate for synthesizing the critical antioxidant glutathione (GSH), which is vital for maintaining redox homeostasis and function in aged immune cells, particularly given the significant increase in oxidative stress during aging [106]. Following prolonged or exhaustive exercise, plasma Gln concentrations often decline significantly (by 20–30%), creating a “post-exercise glutamine depletion window” [107,108]. This reduction in Gln levels can transiently limit the proliferative capacity and function of immune cells such as lymphocytes, which is associated with the transient immunosuppression (e.g., increased susceptibility to upper respiratory tract infections) observed during high-intensity training periods in athletes [109].

Arginine metabolic remodeling: macrophage polarization and T-cell suppression pathways. Its metabolism in immune cells determines functional phenotypes. In the pro-inflammatory pathway, stimulated by IFN-γ and LPS, inducible nitric oxide synthase (iNOS) is highly expressed and utilizes Arg to produce large amounts of cytotoxic nitric oxide (NO) [110]. In the anti-inflammatory/suppressive pathway, stimulated by IL-4/IL-10, arginase 1 (Arg1) is highly expressed and catabolizes Arg into ornithine [111]. More importantly, Arg1 exerts immunosuppressive effects by depleting local arginine required by T cells, thereby inhibiting T-cell proliferation and function via suppression of the T-cell receptor ζ-chain (CD3ζ) and cell cycle arrest [112]. This arginine depletion by Arg1-expressing cells (myeloid-derived suppressor cells [MDSCs], M2 macrophages) is a recognized feature of aging-associated immune dysfunction, where accumulated dysfunctional myeloid populations create arginine-depleted microenvironments that impair T-cell responses.

Acute intense exercise can induce immunosuppression through Arg1 upregulation, whereas long-term, regular, moderate-intensity exercise tends to shape an anti-inflammatory environment [113], improving the overall immune regulatory capacity and T-cell function through mechanisms such as optimizing the Arg1/iNOS balance and reducing myeloid-derived suppressor cell (MDSC) suppressive effects—particularly important for maintaining T-cell function during aging [114]. Importantly, recent evidence reveals that arginine metabolism intersects with tryptophan catabolism (via indoleamine 2,3-dioxygenase 1 [IDO1]) in macrophages: both pathways converge on mTOR and aryl hydrocarbon receptor (AhR) signaling to coordinately regulate macrophage polarization and immune responses [110].

Branched-chain amino acid metabolic remodeling: mTOR-mediated T-cell differentiation and memory formation. Comprising leucine (Leu), isoleucine (Ile), and valine (Val), BCAAs are not only fundamental building blocks for protein synthesis but also important signaling molecules and energy substrates that directly participate in immune cell functional regulation. Leu plays a pivotal role in T-cell fate determination by activating mTORC1 [115]; elevated mTORC1 activity drives effector T-cell (Th1, Th17) differentiation and function, while moderate inhibition favors Treg and memory T-cell formation, which is crucial for maintaining immune homeostasis during aging [116]. Ile and Val serve as important alternative energy sources during the rapid proliferation of activated lymphocytes and can act as precursors for synthesizing key metabolites such as glutamine [104]. Adequate Val supply is a prerequisite for the effective proliferation of aged T cells [102], while Ile participates in regulating B-cell antibody production and maintaining aged macrophage function [117].

Critically, BCAAs modulate immune inflammation not only through mTOR activation but also via AMPK-mediated counter-regulation [101]. Regular exercise, particularly endurance training, significantly upregulates the expression and activity of key BCAA catabolic enzymes (branched-chain aminotransferase [BCAT] and branched-chain α-ketoacid dehydrogenase [BCKDH]) in tissues such as skeletal muscle [118], enhancing the body’s overall capacity to catabolize and utilize BCAAs. This leads to adaptive changes in their circulating concentrations, tissue distribution, and metabolic flux, thereby modulating immune function and delaying immunosenescence.

Collectively, these three amino acids exemplify a nutrient-sensing regulatory system that exercise modulates to support immune function during aging. At the substrate level, exercise optimizes amino acid availability through enhanced tissue utilization and metabolic efficiency. At the signaling level, exercise influences the mTOR-AMPK axis through modulation of BCAA metabolism and availability. At the microenvironmental level, exercise impacts the balance of amino acid-dependent immunosuppressive and pro-inflammatory signals. This creates a coordinated response: exercise-enhanced amino acid metabolism → optimized substrate availability → balanced mTOR/AMPK signaling → preserved immune cell function → delayed immunosenescence [101,117]. Unlike pharmacological mTOR inhibitors (e.g., rapamycin), which globally suppress immune function, exercise achieves metabolic modulation through physiological adaptation that maintains the capacity for protective immune responses while reducing chronic inflammation. These findings underscore that amino acid metabolism represents an important mechanism through which exercise maintains immune homeostasis during aging.

### 3.2. Bidirectional Organ–Immune Crosstalk Networks

#### 3.2.1. The Muscle–Immune Axis

Contracting skeletal muscle functions as an endocrine organ that synthesizes and secretes a diverse array of signaling molecules, collectively termed myokines, which constitute a critical bridge for muscle–immune communication and form an immune regulatory network with synergistic effects [119,120]. The muscle–immune relationship is not unidirectional but represents a dynamic, bidirectional crosstalk: while exercise-induced myokines (including heat shock proteins, interleukins, and metabolic regulators) reprogram immune cell phenotypes and suppress inflammaging, immune-derived inflammatory cytokines (TNF-α, IL-6, IL-1β) conversely drive muscle wasting in aging, creating a vicious cycle that accelerates sarcopenia and immunosenescence [17,121]. Understanding this bidirectional axis reveals why breaking the cycle through exercise—which simultaneously reduces immune-derived catabolic signals while enhancing muscle-derived protective myokines—is uniquely effective at delaying aging. This section integrates the muscle-to-immune (anabolic) and immune-to-muscle (catabolic) pathways, highlighting how exercise-induced molecular chaperones (heat shock proteins) and myokines (IL-6, IL-15, irisin, FGF21) act as a unified “stress response–endocrine network” that recalibrates this axis to favor healthy aging.

Exercise-induced stress response and immune modulation: the HSP–myokine continuum. The aging process is frequently accompanied by protein homeostasis dysregulation. Acute exercise, especially high-intensity or eccentric exercise, induces increased protein degradation and structural damage in skeletal muscle tissue, triggering a stress response that leads to the upregulated expression and potential release of molecular chaperones such as heat shock proteins (HSPs). As intracellular molecular chaperones, HSPs are responsible for the proper protein folding, transport, repair, and degradation of damaged proteins to maintain protein homeostasis, which is crucial for counteracting aging-associated protein misfolding and aggregation [122]. Following a single bout of exhaustive or high-intensity exercise, HSP70 mRNA and protein levels are significantly elevated in human peripheral blood mononuclear cells (PBMCs) and skeletal muscle [123]. This adaptive response helps protect cells from damage and maintain function, which is particularly important for the survival and function of aged lymphocytes under stress conditions [124].

Under specific conditions, such as cell necrosis or stress, HSPs can be released extracellularly [125]. Extracellular HSPs (eHSPs), particularly eHSP70 and eHSP60, act as damage-associated molecular patterns or “alarmins,” activating innate immunity by binding to receptors on immune cell surfaces (TLR2, TLR4, CD91, LOX-1, etc.) [126,127]. For example, eHSP70 stimulates monocytes/macrophages and dendritic cells (DCs) to produce pro-inflammatory cytokines (TNF-α, IL-1β, IL-6) and chemokines while promoting DC maturation and antigen presentation [128], facilitating clearance of cellular debris generated by exercise-induced damage and initiating tissue repair. Critically, this transient pro-inflammatory signal serves a distinct purpose from chronic inflammaging: it activates repair programs and primes immune cells for enhanced pathogen responses [129]. However, excessive or sustained eHSP signaling may exacerbate aging-associated inflammation [130]. Long-term regular exercise training enhances stress tolerance in aged cells by elevating basal intracellular HSP levels and stress-inducible capacity while modulating eHSP release patterns or downstream signaling pathways to control post-exercise inflammatory responses and promote effective pathogen responses in aging organisms [131,132]. Notably, resistance exercise is associated with concurrent elevations in serum HSP70 and anti-inflammatory cytokine IL-10, suggesting coordinated stress-responsive anti-inflammatory signaling [132]. Long-term Tai Chi practice can increase serum HSP70 levels in elderly individuals and is associated with improved immune function, providing protein homeostasis support for delaying immunosenescence.

Myokine-mediated immune reprogramming: a multi-target network. Beyond HSPs, skeletal muscle simultaneously secretes a constellation of myokines that collectively shape anti-aging immune balance through direct immunomodulation (polarizing macrophages and T cells), metabolic regulation (optimizing glucose/lipid metabolism to reduce metabolic stress on immune cells), and systemic anti-inflammatory signaling (suppressing NF-κB and NLRP3 pathways) [119,120].

IL-6: context-dependent master regulator. IL-6 is the most extensively studied myokine. Exercise-induced IL-6 primarily originates from contracting muscle fibers, and its plasma concentration can increase dramatically during prolonged exercise (up to >100-fold above baseline), typically independent of or preceding elevations in pro-inflammatory markers such as TNF-α [133]. Crucially, this exercise-induced IL-6 exhibits an anti-inflammatory profile that is fundamentally distinct from the chronically elevated IL-6 observed in aging and obesity [120]. Exercise-induced IL-6 significantly induces the production of anti-inflammatory cytokines (IL-10) and inhibitors (IL-1ra) while suppressing the synthesis of key pro-inflammatory cytokines (TNF-α) [134], serving as a central molecule that limits potential exercise-induced inflammation and mediates systemic anti-inflammatory effects, thereby helping to alleviate aging-associated, chronic low-grade inflammation. This context dependency is critical: genetic studies confirm that muscle-derived IL-6 is indispensable for mediating benefits such as adipose tissue lipolysis and anti-inflammatory signaling, contrasting sharply with the pathological effects of chronic, systemic IL-6 elevation [120].

IL-15: NK cell and T-cell survival factor. IL-15 is stably expressed in muscle and is critical for the development, survival, and functional maintenance of natural killer (NK) cells and CD8+ T cells (key anti-tumor and antiviral immune cells), whose function gradually declines during aging [135,136]. By synergistically enhancing NK cell cytotoxicity and trafficking alongside exercise-induced IL-6 [120], IL-15 provides essential cellular support for immune surveillance in aging organisms. Additionally, IL-15 improves skeletal muscle oxidative metabolism and glucose uptake, creating a metabolic environment that favors immune cell function.

Irisin (generated by FNDC5 cleavage, with exercise being its primary inducer), beyond its metabolic regulatory effects (promoting adipose browning), also possesses direct anti-inflammatory activity. It suppresses aging-associated excessive macrophage activation by promoting M2 polarization [119]. Furthermore, irisin enhances the transcriptional activity and suppressive function of regulatory T cells (Tregs) by upregulating Foxp3 expression [120], which is crucial for maintaining immune tolerance in aging. Notably, irisin also alleviates oxidative stress-induced endoplasmic reticulum stress (ERS) and apoptosis in macrophages, protecting these cells from age-related dysfunction [137].

FGF21 and BDNF: convergent anti-inflammatory pathways. The exercise-induced skeletal muscle production of fibroblast growth factor 21 (FGF21) acts directly on macrophages through specific receptor pathways (FGFR1-Klotho-STAT5), inhibiting the NF-κB signaling pathway and reducing pro-inflammatory cytokine (IL-6 and TNF-α) secretion while promoting anti-inflammatory cytokine (IL-10) expression [138]. This direct targeting of the NF-κB pathway represents a convergent node where multiple myokines coordinate to suppress the master regulator of inflammaging. Brain-derived neurotrophic factor (BDNF), with skeletal muscle being one source of its exercise-induced release, in addition to neuroprotective and energy metabolic effects, can also modulate aging-associated inflammatory processes by influencing immune cell function or metabolic status [139]. BDNF promotes fatty acid oxidation and maintains mitochondrial quality control in aged muscle, which indirectly supports immune cell metabolism [120].

Immune-derived cytokines drive muscle wasting in aging. While the above discussion highlights the beneficial effects of muscle-derived signals on immunity, the reverse pathway—immune-to-muscle—represents a major driver of sarcopenia and functional decline in aging [17,121]. Chronic low-grade inflammation (inflammaging), characterized by elevated circulating levels of pro-inflammatory cytokines (IL-6, TNF-α, IL-1β), directly induces muscle catabolism through multiple mechanisms: (1) Ubiquitin–proteasome pathway activation: Elevated IL-6 and TNF-α upregulate ubiquitin ligases (e.g., MuRF1, atrogin-1) and proteasome activity, leading to accelerated muscle protein degradation [121]. Clinical studies have shown that increased ubiquitin protein and mRNA levels are linked to elevated IL-6 expression in sarcopenic individuals. (2) NF-κB-mediated muscle atrophy: pro-inflammatory cytokines activate the NF-κB pathway, which drives the expression of atrophy-related genes (atrogenes) and contributes to progressive muscle loss and functional decline with aging [17,121]. (3) Cortisol amplification: IL-6 and TNF-α induce the age-related upregulation of 11βHSD1 (based on in vitro investigations), thereby amplifying the synthesis of cortisol and contributing to muscle catabolism in sarcopenia [17,121]. (4) Macrophage-mediated muscle damage: Aged muscle exhibits the increased infiltration of pro-inflammatory M1 macrophages, which produce TNF-α and IL-6 locally. Single-cell RNA sequencing reveals that aged muscle macrophages express elevated pro-inflammatory and senescence-related markers, impairing their capacity to support satellite cell activation and muscle regeneration [17,121]. Resistance training can shift this balance: a total of 14 weeks of progressive heavy resistance training increases anti-inflammatory M2 macrophages (CD11b+/CD206+) in the skeletal muscle of older individuals, promoting repair over destruction. (5) T-cell dysfunction: Age-related decreases in regulatory T cells (Tregs) in muscle are associated with impaired muscle regeneration. Deficiency in IL6Rα on T cells exhibits deficiencies in muscle Tregs during exercise, resulting in a more pronounced decline in muscle mass [17,121].

This bidirectional framework reveals a self-amplifying pathological loop in aging: chronic inflammation (↑ TNF-α, IL-6 from immune cells) → muscle wasting (↓ muscle mass ↓ myokine secretion) → further immune dysfunction (↓ Tregs ↑ M1 macrophages) → exacerbated inflammation. Exercise uniquely disrupts this cycle at multiple nodes simultaneously:

Muscle-to-immune: ↑ exercise-induced IL-6, IL-15, irisin, FGF21 → M2 macrophage polarization, Treg enhancement, NK cell activation → ↓ systemic TNF-α, IL-1β.

Immune-to-muscle: ↓ chronic IL-6/TNF-α → ↓ ubiquitin–proteasome activity ↓ NF-κB activation → preserved muscle mass → sustained myokine production.

Metabolic recalibration: ↑ irisin, BDNF → adipose browning ↑ insulin sensitivity → ↓ metabolic stress on immune cells → ↓ inflammaging.

Notably, resistance training is particularly effective at reducing inflammatory cytokine levels (specifically IL-6) in sarcopenic participants [17], demonstrating that this exercise modality can be tailored to target specific populations.

By simultaneously enhancing muscle-derived protective myokines and suppressing immune-derived catabolic cytokines, exercise transforms the muscle–immune axis from a vicious cycle (inflammation → sarcopenia → immunosenescence) into a virtuous cycle (myokine secretion → immune rejuvenation → muscle preservation) [119,121]. These coordinated actions are crucial for delaying immunosenescence, maintaining immune homeostasis, and promoting adaptive immunity. Furthermore, emerging evidence suggests that the efficacy of this bidirectional recalibration may depend on the exercise modality: resistance training preferentially targets macrophage polarization and reduces local muscle inflammation, while endurance training enhances systemic myokine release and metabolic benefits.

#### 3.2.2. The Gut Microbiota–Immune Axis

As the “second genome” of the human body, the gut microbiota establishes a bidirectional communication network with the immune system through its structural composition and metabolic products, playing a critical role in regulating immune homeostasis. However, this relationship is not unidirectional: while gut dysbiosis drives immune dysfunction and inflammaging, immune-derived inflammatory mediators conversely disrupt intestinal barrier integrity and exacerbate microbial imbalance, creating a self-amplifying pathological loop [140,141]. With advancing age, alterations in the gut microbiota structure not only lead to chronic low-grade inflammation (inflammaging) but also accelerate immunosenescence by affecting immune cell differentiation, activation, and function.

Aging-associated gut dysbiosis and immune abnormalities. Aging-associated studies have shown that gut microbiota α-diversity is significantly reduced with an increased abundance of pro-inflammatory Proteobacteria (major members of Gram-negative bacteria) and a decreased abundance of beneficial bacterial groups with anti-inflammatory effects, including Firmicutes, Clostridiaceae, and Lachnospiraceae [142]. This microbial dysbiosis induces immune abnormalities and triggers peripheral chronic inflammatory responses. Gut microbiota disruption leads to intestinal barrier dysfunction, resulting in “leaky gut”. The intestinal barrier, composed of the mucus layer, intestinal epithelium, and lamina propria, is crucial for maintaining immune homeostasis. Pathogenic bacteria (such as Bacteroides fragilis, Escherichia coli, and Salmonella) can disrupt tight junction proteins and increase intestinal permeability [143,144]. Leaky gut not only causes gut microbiota translocation and leakage of harmful metabolites (such as lipopolysaccharide [LPS] and bacteria-derived amyloid) but also leads to polymorphonuclear leukocyte migration from systemic circulation to the intestinal mucosa, inducing inflammatory responses [145]. Gut-derived pro-inflammatory factors can enter the circulatory system and promote systemic inflammatory responses [146].

The gut microbiota regulates systemic immune function through multiple mechanisms. Research demonstrates that the gut microbiota not only regulates peripheral immune cells but also influences the development and function of central lymphoid organs [147], suggesting its important role in immune regulation. Intestinal inflammation can enhance the migratory capacity of peripheral immune cells, promoting their recruitment to tissues with elevated oxidative stress and affecting systemic immune function through cascade effects [148]. More importantly, sustained antigenic stimulation and inflammatory signals resulting from gut dysbiosis can promote the excessive depletion of naive T cells, accelerate their conversion to aging-associated effector/memory phenotypes, and upregulate the expression of inhibitory receptors such as PD-1, exacerbating T-cell exhaustion [149]. The gut microbiota and its metabolites can directly or indirectly influence dendritic cells and macrophages through epithelial cell intervention, with regulation occurring via epigenetic mechanisms [150,151,152]. Regulatory T cells (Tregs) can also be induced by gut microbiota metabolites [153,154]. The gut microbiota can also induce the maturation of B cells and alter their immunoglobulin subtypes [155,156]. Therefore, gut microbiota dysbiosis induces immune abnormalities and triggers peripheral inflammatory responses, thereby affecting systemic immune function.

Immune-derived mediators disrupt gut homeostasis in aging. While the above discussion highlights how gut dysbiosis impairs immunity, the reverse pathway—immune-to-gut—represents a major driver of intestinal barrier dysfunction and accelerates the progression of aging-associated gut pathology [141,157]. Aging is associated with the chronic elevation of pro-inflammatory cytokines (TNF-α, IFN-γ, IL-1β, IL-6) that directly compromise intestinal epithelial barrier function through multiple mechanisms [140,141]: (1) Tight junction disruption by IFN-γ and TNF-α: Age-related increases in circulating and mucosal IFN-γ directly downregulate tight junction proteins (claudins, occludin, ZO-1), increasing intestinal permeability [141]. Studies in aged mice demonstrate that IFN-γ-mediated barrier dysfunction facilitates bacterial translocation and the systemic dissemination of microbial products, perpetuating inflammaging [141]. TNF-α synergistically amplifies this effect by activating myosin light chain kinase (MLCK), which phosphorylates tight junction proteins and disrupts their structural integrity [158]. (2) TNF-driven intestinal inflammation: Aging is characterized by elevated intestinal TNF production, which drives local inflammation and epithelial cell apoptosis [140]. Importantly, the genetic or pharmacological ablation of TNF signaling in aged mice restores intestinal barrier integrity and reduces systemic inflammation, demonstrating a direct causal role of immune-derived TNF in age-related gut dysfunction [140]. (3) IL-1β-mediated barrier dysfunction: The age-related activation of the NLRP3 inflammasome in intestinal macrophages leads to excessive IL-1β production, which impairs epithelial tight junction assembly and promotes intestinal permeability [157]. This process is partially microbiota-independent, as aged germ-free mice still exhibit elevated IL-1β levels and barrier dysfunction [157]. (4) Treg dysfunction and loss of immune tolerance: Age-related decreases in colonic regulatory T cells (Tregs) result in the inadequate suppression of effector T-cell responses and reduced IL-10 production, failing to counterbalance pro-inflammatory cytokines [159]. This loss of mucosal immune tolerance perpetuates epithelial damage and creates a permissive environment for pathobiont expansion [159]. (5) Feed-forward loop: immune-mediated dysbiosis: critically, this immune-driven barrier dysfunction subsequently alters the microbiota composition, enriching pro-inflammatory taxa (Proteobacteria) and depleting SCFA-producing bacteria (Firmicutes, Lachnospiraceae), which further exacerbates inflammation and creates a self-amplifying vicious cycle [140,141].

Breaking the vicious cycle through exercise. This bidirectional framework reveals a self-amplifying pathological loop in aging: gut dysbiosis (↓ SCFA-producing bacteria ↑ LPS) → systemic inflammation (↑ TNF-α, IFN-γ, IL-1β) → intestinal barrier dysfunction (↓ tight junctions, ↑ permeability) → microbial translocation and dysbiosis → exacerbated inflammation [140,141,157]. Exercise uniquely disrupts this cycle at multiple nodes simultaneously [160]:

Gut-to-immune: ↑ SCFA-producing bacteria (Firmicutes, butyrate-producers) → ↓ LPS ↑ Tregs, ↓ systemic TNF-α/IL-6.

Immune-to-gut: ↓ pro-inflammatory cytokines (TNF-α, IFN-γ, IL-1β) → ↑ tight junction protein expression ↓ intestinal permeability → ↓ bacterial translocation.

Metabolic recalibration: ↑ SCFAs (butyrate, acetate) → GPR43/GPR109A activation → NF-κB and NLRP3 suppression → ↓ intestinal inflammation.

Exercise reshapes the gut microbiota structure. Studies have confirmed that exercise of varying intensities can reshape the gut microbiota structure. Four weeks of moderate-intensity treadmill exercise significantly reversed the reduction in the Firmicutes abundance and the increase in the Bacteroidetes abundance, decreasing the Bacteroidetes/Firmicutes ratio [161]. Sixteen weeks of voluntary wheel running significantly reduced the abundance of pro-inflammatory bacterial groups, such as Proteobacteria and Tenericutes, while increasing the abundance of beneficial bacteria, such as Allobaculum [162]. High-intensity interval exercise significantly increased the relative abundance of butyrate-producing bacterial groups, including Eubacterium, Roseburia, and Clostridium, while suppressing Bacteroides fragilis, which damages intestinal mucosal health [162]. Butyrate-producing bacterial groups such as *Butyrivibrio proteoclasticus*, *Marvinbryantia formatexigens*, and *Roseburia* spp. were significantly increased. Importantly, exercise-induced shifts in microbiota composition are associated with increased microbial diversity and the enhanced production of beneficial metabolites, which collectively contribute to improved gut barrier function and reduced systemic inflammation in older adults.

Exercise protects intestinal barrier integrity. Scientific and rational physical activity can prevent intestinal inflammatory diseases. Studies have shown that voluntary wheel running attenuates inflammatory gene expression in the distal colon, significantly reduces the levels of pro-inflammatory cytokines, including IL-1β and TNF-α, decreases diarrhea incidence, and inhibits secondary infection by Escherichia coli, conferring protective effects against experimental colitis [163,164,165]. Swimming for 30 min/day enhances the intestinal antimicrobial capacity and alleviates chronic stress-induced intestinal barrier dysfunction by increasing the tissue expression of the α/β-defensins and RegIIIβ/γ genes and antimicrobial peptide production [166]. Four weeks of moderate-intensity treadmill exercise can also elevate intestinal tight junction protein expression levels, improve intestinal barrier damage, and reduce the serum LPS content [167]. Mechanistically, exercise reduces circulating levels of barrier-disrupting cytokines (TNF-α, IFN-γ) while promoting the expression of tight junction proteins (ZO-1, occludin, claudins), thereby restoring barrier integrity [160]. Even single bouts and short-term acute exercise promote the expression of anti-inflammatory cytokines IL-6 and IL-10 in intestinal lymphocytes [168,169].

Exercise regulates gut microbiota metabolites. Exercise improves peripheral chronic inflammatory responses by regulating the gut microbiota metabolite kynurenine (Kyn). Its secondary metabolite kynurenic acid (Kyna) can suppress LPS-induced inflammatory responses in monocytes and macrophages and control cytokine release from invariant natural killer T (iNKT) cells [170]. Simultaneously, Kyna can activate the aryl hydrocarbon receptor (AhR) on immune cells, promoting immune tolerance [171]. Eight weeks of voluntary wheel running can activate skeletal muscle peroxisome proliferator-activated receptor γ coactivator 1α1 (PGC-1α1), increase kynurenine aminotransferase (KAT) gene expression, and promote Kyn-Kyna conversion, thereby inhibiting Kyn accumulation and improving inflammatory responses [172].

Short-chain fatty acids (SCFAs) are among the most important metabolites of the gut microbiota and play critical roles in immune cell development and functional regulation. Acetate is essential for immune cell maturation and maintenance of metabolic homeostasis; germ-free mice or lower gut microbiota diversity can lead to immune cell developmental defects, resulting in impaired immune responses [147]. Butyrate functions as an anti-inflammatory agent through its role as a histone deacetylase inhibitor. Six weeks of voluntary wheel running effectively increased the butyrate/acetate ratio in the mouse intestine [173]. Compared to sedentary populations, professional rugby players showed significantly elevated fecal SCFAs, including acetate and butyrate [174]. Exercise interventions in older adults have been shown to increase the abundance of SCFA-producing bacteria and elevate circulating SCFA levels, which contribute to improved metabolic health and reduced systemic inflammation [175]. Different exercise intensities and modalities exhibit distinct effects on SCFA regulation. Rats subjected to combined aerobic and resistance exercise intervention showed decreased fecal acetate levels with no significant change in butyrate [176], whereas exhaustive exercise in rats decreased the intestinal acetate proportion while increasing the butyrate proportion [177]. This suggests that scientific and rational exercise can optimize the SCFA composition (e.g., by increasing the relative proportion of butyrate), thereby enhancing anti-inflammatory effects while maintaining the metabolic requirements for immune cell function.

Exercise improves local intestinal immune function. Additionally, exercise can improve the microbiota structure by regulating local intestinal immune function. Compared to sedentary control mice, mice undergoing long-term moderate-intensity exercise showed elevated intestinal IgA levels, the upregulated gene expression of TGF-β, IL-6, TNF-α, IL-4, and IL-10, downregulated IL-2 gene expression, and reduced numbers of B cells and CD4+ T cells. The exercise-induced elevation of intestinal IgA levels can enhance immune function, improve resistance to intestinal pathogen infection, enhance discrimination of commensal microbiota and protection of their colonization, and improve the microbiota structure [178,179]. Comprehensive studies demonstrate that 12 weeks of moderate-intensity treadmill exercise effectively increases gut microbiota α-diversity, enhances enrichment of beneficial bacterial groups, improves intestinal barrier permeability, suppresses intestinal inflammation, reverses intestinal pathology, and reduces systemic inflammatory responses [180].

By simultaneously enhancing gut-derived protective metabolites (SCFAs, Kyna) and suppressing immune-derived barrier-disrupting cytokines (TNF-α, IFN-γ, IL-1β), exercise transforms the gut–immune axis from a vicious cycle (dysbiosis → inflammation → barrier dysfunction → microbial translocation → exacerbated inflammation) into a virtuous cycle (microbial diversity → SCFA production → immune tolerance → barrier integrity → reduced systemic inflammation) [140,160]. This indicates that exercise can improve immune function and suppress dysbiosis-induced inflammatory responses from three aspects—the gut microbiota, the peripheral immune system, and systemic immune regulation—by improving the gut microbiota structure and metabolic products via both microbiota-dependent and microbiota-independent immune pathways, thereby ameliorating the occurrence and progression of immunosenescence-associated pathology.

### 3.3. Remodeling of Innate and Adaptive Immune Cells

#### 3.3.1. Modulation of Innate Immune Cells

The aging of the innate immune system is primarily characterized by “inflammaging” and the functional decline in neutrophils, macrophages, and natural killer (NK) cells. Research indicates that exercise can improve innate immune function by remodeling the inflammatory environment in the elderly. Specifically, moderate-intensity aerobic exercise has been shown to significantly reduce C-reactive protein (CRP) levels and enhance the respiratory burst capacity of neutrophils, thereby improving their bactericidal activity [181]. Intervention studies in older adults further confirm that regular exercise regulates oxidative stress and inflammatory pathways by upregulating superoxide dismutase (SOD) and reducing malondialdehyde (MDA) levels. This is manifested as a decrease in pro-inflammatory cytokines such as TNF-α and IL-6, thereby delaying the progression of immunosenescence [182,183]. However, the exercise intensity represents a critical variable, as high-intensity exercise may induce oxidative stress and inflammatory imbalance [184], underscoring the importance of moderate exercise.

At the cellular level, moderate exercise also exerts a significant enhancing effect on macrophages and NK cells. Mcfarlin et al. confirmed that long-term resistance training increases resting NK cell activity in elderly women, effectively counteracting the age-related decline in immune surveillance capabilities against tumors and infections [185]. Nevertheless, the risk of overtraining warrants attention; Kawada et al. noted that high-frequency, high-intensity resistance training might conversely reduce NK cell activity and accelerate immunosenescence [186], highlighting the necessity of scientifically monitoring exercise loads.

Furthermore, the amelioration of innate immunity by exercise involves regulation at deeper molecular levels. A 16-week combined exercise (aerobic + resistance) study in the elderly showed that exercise not only downregulated TNF-α and upregulated the anti-inflammatory cytokine IL-10 but also involved the activation of the AMPK signaling pathway and epigenetic regulatory mechanisms [187]. Simultaneously, exercise intervention can effectively reverse the exacerbated expression of immunosenescence markers associated with obesity in middle-aged individuals [187]. Some resistance training studies have also observed increases in IL-2 and IFN-γ levels alongside improvements in systemic immune status [188], suggesting that exercise provides a crucial pathway for delaying overall immunosenescence by improving the innate immune environment and the function of key immune cells.

#### 3.3.2. Mitigation of Conventional T-Cell Senescence

The decline in T-cell immune function, characterized by the contraction and dysfunction of the T-cell repertoire, is a hallmark of immunosenescence. With advancing age, lymphoid progenitors exhibit differentiation defects, leading to a dual decline in both the quantity and quality of T cells. This is primarily manifested as the depletion of the naive T-cell reserve and the accumulation of immunosenescent, terminally differentiated T cells [189]. Exercise remodels conventional T cells through a coordinated process involving mobilization, selective apoptosis, and regeneration. The core mechanism is hypothesized to be the “vacating space” theory: exercise induces the apoptosis of terminally differentiated senescent T cells, thereby creating immunological space for the output of naive T cells from the bone marrow and thymus [190,191,192]. This mechanism of cellular renewal not only restores the diversity of the peripheral T-cell repertoire but also enhances the immune response to vaccination [193,194]. Furthermore, it is considered a potential mechanism by which exercise improves anti-tumor immune surveillance [195].

Thymic involution is a fundamental cause of T-cell immunosenescence. Research indicates that long-term moderate-intensity exercise can delay thymic atrophy and may even partially restore thymic hematopoiesis [196]. A landmark study by Duggal et al. demonstrated that maintaining high levels of physical activity throughout adulthood or lifelong can ameliorate reduced thymic output, a key feature of immunosenescence [190].

Several potential mechanisms may underlie the exercise-induced enhancement of thymic T-cell output. First, exercise may modulate systemic and local growth factors critical for thymic function, including insulin-like growth factor-1 (IGF-1) and interleukin-7 (IL-7), both of which promote thymocyte proliferation, survival, and differentiation [197]. Second, exercise-induced reduction in chronic low-grade inflammation may protect thymic epithelial cells (TECs) from inflammaging-associated damage, thereby preserving their capacity to support T-cell development and positive/negative selection [190]. Third, regular physical activity may enhance thymic vascularization and oxygen delivery, counteracting age-related thymic adipose involution and improving the microenvironmental niche for hematopoietic progenitor seeding and T-cell maturation. Fourth, exercise may reduce thymic fat infiltration, a hallmark of age-related thymic degeneration, thereby restoring the functional thymic tissue volume [190]. Exercise improves the thymic microenvironment, thereby enhancing the production of naive T cells and increasing the efficiency of their export into the peripheral circulation [160]. Such adaptive changes induced by regular exercise help rebalance the immune regulatory network in the aging organism and maintain the regenerative capacity of the immune system [198].

Beyond regulating cell numbers and proportions, exercise significantly improves the functional quality of T cells. Chronic exercise training has been shown to significantly increase the proportion of activated CD4+ T cells in peripheral blood and restore their proliferative capacity in vitro [199]. Regarding CD8+ T cells, exercise not only promotes their expansion in vitro but also enhances their sensitivity in recognizing tumor or viral antigen peptides. Additionally, exercise increases T-cell receptor (TCR) repertoire diversity, thereby elevating the specific immune response capability against novel pathogens [200,201,202,203]. The mechanism underlying the exercise-induced enhancement of TCR diversity appears to involve both the augmented thymic output of newly generated naive T cells—each carrying unique, randomly recombined TCR sequences generated through VDJ recombination—and the selective apoptotic clearance of clonally expanded, terminally differentiated senescent T cells that dominate the aged TCR repertoire [191,204]. By simultaneously increasing the influx of diverse naive T cells and reducing the oligoclonal expansion of exhausted effector T cells, exercise effectively “resets” the peripheral TCR landscape toward a more polyclonal, youthful configuration. This dual mechanism—thymic rejuvenation and peripheral editing—collectively restores immune repertoire breadth and enhances the capacity to recognize novel antigens [205,206].

Current evidence suggests that exercise-induced T-cell modulation is intensity- and time-dependent. Regarding chronic effects, Spielmann et al. found that higher aerobic fitness (VO_2max_) is significantly associated with a lower proportion of senescent T cells (CD28−/CD57+) in human peripheral blood [191]. Similarly, Minuzzi et al. confirmed that long-term training improves the mobilization capacity and response efficiency of aging lymphocytes [192]. Regarding acute effects and intensity differences, Kruger et al. noted that acute high-intensity interval exercise (HIIT) induces a significant redistribution of T-cell subsets in peripheral blood: a dramatic increase in CD3+, CD4+, and CD8+ T-cell counts immediately post-exercise (lymphocytosis), followed by a decline to below baseline levels 3 h post-exercise (lymphocytopenia). Notably, this process is accompanied by the preferential apoptosis of highly differentiated (terminally differentiated) T cells [196,207], acting as a mechanism for the selective clearance of dysfunctional cells.

#### 3.3.3. Mechanisms of Exercise-Induced Apoptosis in Terminally Differentiated Senescent T Cells

The preferential clearance of senescent T cells mentioned above involves multiple convergent apoptotic pathways that selectively target highly differentiated cells while preserving functional lymphocytes [196,207,208]. Understanding these mechanisms is critical for optimizing exercise interventions.

(1) The Fas/FasL-mediated extrinsic pathway. Evidence suggests that terminally differentiated senescent T cells (CD28^−^CD57^+^) express elevated levels of the Fas (CD95) death receptor. Exercise may acutely increase circulating Fas ligand (FasL) levels and, through catecholamine-mediated β2-adrenergic signaling [184], potentially enhance apoptotic susceptibility in senescent cells.

(2) Oxidative stress and mitochondrial pathways. High-intensity exercise transiently increases reactive oxygen species (ROS) production [209]. Senescent T cells exhibit accumulated mitochondrial dysfunction and reduced antioxidant capacity, making them more vulnerable to oxidative stress-induced apoptosis. In contrast, naive and functional memory T cells are protected by the higher expression of anti-apoptotic proteins (such as Bcl-2) and more robust antioxidant defenses [196,207,208].

(3) Glucocorticoid-mediated apoptosis. Exercise acutely elevates circulating glucocorticoids (cortisol), which can induce lymphocyte apoptosis through the transcriptional regulation of pro-apoptotic and anti-apoptotic genes. Senescent T cells may exhibit differential sensitivity to this pathway, contributing to the post-exercise lymphocytopenia observed several hours after high-intensity exercise [196,207,208].

(4) Activation-induced cell death (AICD). Exercise-induced activation signals may trigger AICD in senescent cells that have exhausted their replicative potential and possess shortened telomeres, acting as a quality control mechanism [196,207,208].

Through these selective mechanisms, exercise reduces the homeostatic burden on the T-cell compartment, potentially allowing for the expansion of newly generated naive T cells from the thymus [190,191,192].

#### 3.3.4. Remodeling of Innate-like Unconventional T Cells

Understanding immune aging requires integrating both conventional αβ T cells and unconventional T cells (including γδ T cells, iNKT cells, and MAIT cells). These cells bridge innate and adaptive immunity, recognizing non-peptide antigens and exhibiting rapid “innate-like” responses [210]. Collectively, they comprise approximately 10–30% of the T-cell compartment in adults, particularly at barrier sites like the gut and liver:

(1) γδ T cells. Aging leads to a progressive decline in “innate-like” Vγ9Vδ2^+^ γδ T cells, while “adaptive-like” Vδ1^+^ cells may expand due to CMV infection [210]. Exercise mobilizes γδ T cells to blood via β2-adrenergic receptor signaling. Specifically, acute graded exercise preferentially mobilizes effector Vγ9Vδ2^+^ cells with enhanced cytotoxic phenotypes, which may represent a mechanism for improved anti-tumor surveillance [211].

(2) iNKT cells. These cells (invariant natural killer T cells) exhibit age-related functional shifts from Th1 to Th2 cytokine profiles and gender-dependent declines in frequency [210]. While specific exercise data is emerging, the potential for exercise to modulate the circulating pool of these cells remains an important area for future research.

(3) MAIT cells. Mucosal-associated invariant T (MAIT) cells are highly responsive to exercise. Acute moderate-intensity aerobic exercise substantially increases circulating MAIT cell counts, with the preferential mobilization of CD8^+^ subsets expressing chemokine receptors CCR4, CCR5, and CCR6. Critically, exercise-mobilized MAIT cells exhibit enhanced functional capacity, showing increased TNFα and IFN-γ expression upon stimulation. Long-term training may help rescue age-related impairments in MAIT cell mobilization [212].

Exercise promotes adaptive immune rejuvenation through a unified framework: it mobilizes highly differentiated effector cells (both conventional and innate-like) for immediate defense, selectively induces apoptosis in senescent cells to create space, and supports thymic output. This integrated remodeling transforms the aging T-cell compartment into a more rejuvenated and responsive repertoire.

### 3.4. Molecular and Epigenetic Mechanisms

#### 3.4.1. Circulating Mitochondrial DNA and Inflammatory Attenuation

A core hallmark of immunosenescence is chronic low-grade inflammation, termed “inflammaging”. Elevated levels of circulating mitochondrial DNA (cf-mtDNA), a potent damage-associated molecular pattern (DAMP), are closely linked to aging-associated pathology by triggering sterile inflammation. Regular exercise has been proven to effectively lower circulating mtDNA levels, thereby ameliorating chronic inflammation. Research indicates that professional male volleyball players exhibit significantly lower resting plasma cf-mtDNA compared to that of sedentary controls [213], suggesting an association between high-level physical activity and reduced inflammatory triggers. Acute exercise also demonstrates immediate benefits: a single bout of aerobic exercise (60% VO_2max_) significantly reduced plasma cf-mtDNA levels at 54 and 90 min post-exercise [214].

Beyond direct clearance, chronic aerobic exercise remodels the gene expression profile of peripheral leukocytes, downregulating pro-inflammatory genes while upregulating anti-inflammatory genes [215,216]. This process involves multiple pathways, including reducing the count of pro-inflammatory monocyte subsets, downregulating the TLR4 expression on monocytes, and stimulating the release of anti-inflammatory myokines from skeletal muscle [217]. Mechanistically, it has been shown that 6 weeks of voluntary wheel running reduces leptin secretion from adipose tissue, which, in turn, increases the CXCL12 expression in bone marrow stromal cells (LepR+). This suppresses the proliferation of hematopoietic stem and progenitor cells (LSKs), leading to a reduction in pro-inflammatory leukocyte output. Notably, this reduction did not compromise immunity; rather, it enhanced anti-infection capabilities in mice by minimizing inflammation-induced tissue damage [218]. However, the intensity of exercise acts as a “double-edged sword”; short-term high-intensity exercise can induce mitochondrial dysfunction in peripheral leukocytes, accompanied by increased apoptosis and elevated pro-inflammatory mediators [219], underscoring the critical role of moderate intensity in managing immunosenescence.

#### 3.4.2. Autophagy-Mediated Restoration of Immune Cell Function

During aging, autophagic flux in immune cells is impaired, leading to the accumulation of damaged organelles and protein aggregates. Exercise serves as a potent physiological stressor that activates the autophagy system, acting as a “quality control” mechanism. Regardless of the intensity, acute exercise enhances mitophagy in both skeletal muscle and immune cells [220]. This mechanism is directly linked to the reduction in circulating mtDNA: by activating mitophagy, exercise facilitates the timely clearance of damaged mitochondria, preventing mtDNA leakage [214,220]. In models of high-intensity exhaustive exercise, the compensatory activation of mitophagy clears damaged mitochondria and reduces mtDNA release, thereby attenuating the activation of the cGAS-STING pathway and subsequent interferon/inflammatory signaling cascades.

At the molecular level, exercise regulates the autophagy network primarily through the energy sensor AMPK. AMPK phosphorylates ULK1 (at Ser555) and inhibits mTORC1 activity, thereby initiating the autophagy complex [221]. Subsequently, the Beclin-1/VPS34 complex mediates phagophore formation, while LC3-I is converted to lipidated LC3-II via ATG7/3, participating in autophagosome elongation [222]. This cascade is pivotal for clearing oxidative damage products accumulated in senescent immune cells and restoring cellular homeostasis.

#### 3.4.3. Mitochondrial Optimization and Immunometabolic Reprogramming

Dysregulated “immunometabolism”, characterized by declined oxidative phosphorylation (OXPHOS) capacity and excessive ROS production, is a key feature of immunosenescence. Exercise reverses this aging phenotype by promoting mitochondrial biogenesis and metabolic reprogramming. Specifically, exercise upregulates PGC-1α expression via the CaMKKβ and AMPK signaling pathways [223]. As the master regulator, PGC-1α binds with Nuclear Respiratory Factors 1/2 (NRF1/2) to promote Mitochondrial Transcription Factor A (TFAM) expression, driving mtDNA transcription and replication to enhance OXPHOS capacity [224].

Furthermore, exercise-enhanced mitochondrial metabolism supports the transition of immune cells (such as macrophages) towards an anti-inflammatory M2 phenotype and suppresses excessive ROS production [225]. Crucially, enhanced mitochondrial biogenesis [226] and autophagy (e.g., increased LC3 expression) [227] via the PGC-1α/TFAM axis effectively clear damaged mitochondria. This prevents mtDNA leakage into the cytosol, thereby blocking the aberrant activation of the NLRP3 inflammasome.

#### 3.4.4. Epigenetic Reprogramming in Immunosenescence

Immunosenescence is accompanied by cumulative alterations in the epigenetic landscape, resulting in the sustained activation of pro-inflammatory genes and the silencing of immune function genes. Exercise can delay or reverse this process through epigenetic reprogramming. First, by modulating metabolite availability (e.g., Acetyl-CoA) and reducing DAMP release, exercise induces “Trained Immunity.” This mechanism enables senescent innate immune cells to regain a rapid and robust response to pathogens via specific epigenetic modifications [228].

**Figure 2:**
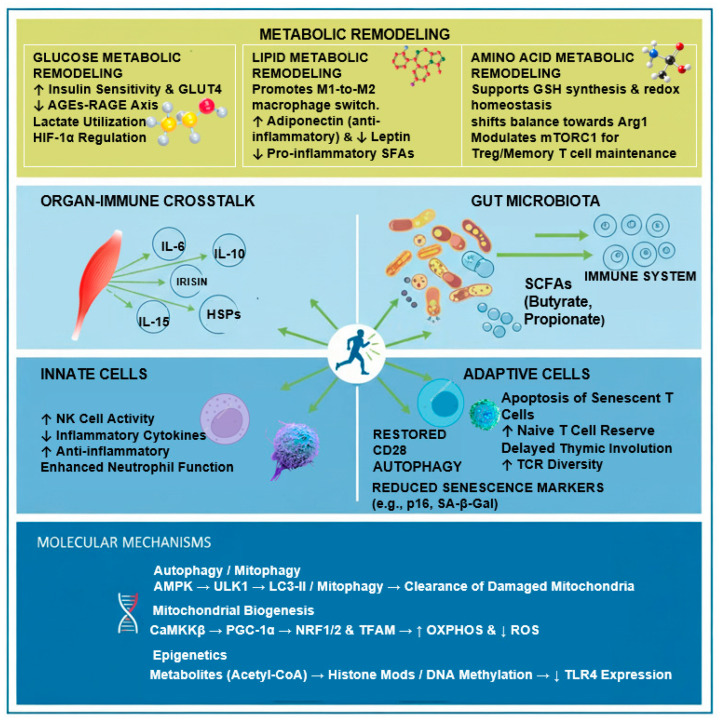
Multidimensional mechanisms by which exercise ameliorates immunosenescence. This schematic illustrates the integrated pathways through which exercise counteracts aging-associated immune dysregulation, spanning the metabolic, systemic, cellular, and molecular levels. (Top) Metabolic Remodeling: exercise corrects immunometabolism by enhancing glucose metabolism (increasing insulin sensitivity and GLUT4 expression, regulating HIF-1α), optimizing lipid metabolism (promoting M1-to-M2 macrophage transition, increasing adiponectin, and reducing pro-inflammatory SFAs), and remodeling amino acid metabolism (maintaining glutamine/arginine balance to support redox homeostasis). (Middle Upper) Organ–Immune Crosstalk: exercise strengthens systemic communication via the muscle–immune axis, where contracting muscles release myokines (e.g., IL-6, IL-15, Irisin) and heat shock proteins (HSPs), and via the gut–immune axis, where the remodeled gut microbiota produces short-chain fatty acids (SCFAs) to modulate immune responses. (Middle Lower) Cellular Remodeling: exercise rejuvenates the immune system by enhancing the activity of innate immune cells and restoring adaptive immune cells (particularly T cells) through the upregulation of costimulatory molecules (e.g., CD28), induction of autophagy, and downregulation of senescence markers (e.g., p16, SA-β-Gal). (Bottom) Molecular Mechanisms: mechanistically, exercise mitigates sterile inflammation by reducing circulating mtDNA and activating three key pathways: (1) autophagy/mitophagy (via the AMPK/ULK1/LC3-II axis) to clear damaged mitochondria; (2) mitochondrial biogenesis (via the CaMKKβ/PGC-1α/TFAM axis) to enhance OXPHOS and reduce ROS; and (3) epigenetic reprogramming (via histone modifications and DNA methylation) to silence pro-inflammatory gene expression (e.g., TLR4). AMPK: AMP-activated protein kinase; HIF-1α: Hypoxia-inducible factor 1-alpha; SCFAs: short-chain fatty acids; GLUT4: Glucose transporter type 4; AGEs/RAGE: advanced glycation end products/receptor for AGEs; SFAs: saturated fatty acids; HDL: high-density lipoprotein; GSH: glutathione; BCAA: branched-chain amino acids; mTORC1: mammalian target of rapamycin complex 1; PGC-1α: peroxisome proliferator-activated receptor gamma coactivator 1-alpha; NRF1/2: nuclear respiratory factor 1/2; TFAM: mitochondrial transcription factor A; LC3-II/ULK1: microtubule-associated protein 1A/1B-light chain 3-II/Unc-51 like autophagy activating kinase 1; SA-β-Gal: senescence-associated beta-galactosidase; OXPHOS: oxidative phosphorylation; mtDNA: mitochondrial DNA; HSPs: heat shock proteins. (Note: ↑ indicates in-crease/upregulation; ↓ indicates decrease/downregulation/impairment).

Second, exercise directly regulates DNA methylation and non-coding RNA networks. Regarding DNA methylation, exercise alters the methylation status of key immune gene promoters (e.g., TLR4, IL-1β) by downregulating DNA methyltransferase (DNMT) activity or modulating Ten-Eleven Translocation (TET) enzymes [229]. For instance, endurance exercise significantly reduces TLR4 expression in monocytes, an anti-inflammatory adaptation closely tied to specific histone modifications and DNA methylation patterns. Additionally, exercise regulates the expression of immune-related microRNAs (e.g., miR-155, miR-21) [222,230], providing fine-tuned post-transcriptional regulation of immune signaling pathways to reverse aging-associated epigenetic anomalies (Figure 2).

## 4. Dose–Response Relationship Between Different Exercise Modalities and Attenuation of Immunosenescence in Older Adults

Before examining the evidence, it is essential to define the criteria typically used to classify exercise intensity in older adult populations, as these definitions vary by modality. For aerobic exercise, intensity is commonly prescribed using the heart rate reserve (HRR), percentage of the maximal heart rate (%HRmax), or rating of perceived exertion (RPE). According to the American College of Sports Medicine (ACSM) guidelines, moderate intensity corresponds to a 40–59% HRR or a 64–76% HRmax, with an RPE of 12–13 on the Borg 6–20 scale. High (vigorous) intensity is defined as a ≥60% HRR or a ≥77% HRmax, with an RPE ≥ 14. For resistance training, intensity is primarily determined by the percentage of one-repetition maximum (%1RM), where 50–69% 1RM is considered moderate and ≥70% 1RM is high intensity. In contrast, mind–body exercises (e.g., Tai Chi, Qigong) are generally categorized as low-to-moderate intensity based on metabolic equivalents (METs, typically 3–6 METs) and subjective effort, although intensity can fluctuate with proficiency. These standardized criteria provide the framework for interpreting the dose–response relationships discussed below [231,232,233,234].

Accumulating evidence suggests that the attenuation of immunosenescence follows a clear dose–response pattern that is jointly determined by the exercise modality, intensity, duration, and cumulative lifetime exposure rather than by any single training component alone. From the perspective of exercise modality, aerobic exercise is currently supported by the most robust evidence. Studies by Woods et al. [235] and Kohut et al. [236] demonstrated that approximately 10 months of moderate-intensity cardiovascular training not only significantly enhanced influenza vaccine antibody responses in older adults but also prolonged serological protection. These findings indicate that when aerobic exercise reaches sufficient duration and frequency, it can partially overcome age-related vaccine hyporesponsiveness, a functional hallmark of immunosenescence.

At the mechanistic level, this functional improvement appears closely linked to the regulation of chronic low-grade inflammation. The systematic review by Tayebi et al. [237] showed that regular aerobic training significantly reduced circulating CRP, TNF-α, and IL-6. Because “inflammaging” is a central driver of immune dysfunction in aging, these data suggest that aerobic exercise may act upstream by dampening inflammatory loading, thereby slowing the progression of immunosenescence rather than merely improving isolated immune outcomes.

Resistance training likewise plays an important role in modulating immunosenescence. The systematic review by Salimans et al. [23] reported that resistance exercise can improve multiple aspects of immune cell function, reduce susceptibility to infections, and enhance vaccine responses. Consistently, the meta-analysis by Kim et al. [238], which included 18 randomized controlled trials, confirmed that moderate-dose resistance training produced a medium effect size reduction in CRP and significantly decreased IL-10 and TNF-α concentrations, with a trend towards reduced IL-6. These findings support the notion that resistance training contributes indirectly to immune aging by improving muscle mass and metabolic health, which, in turn, modulate systemic inflammatory and immune environments.

Importantly, evidence increasingly indicates that combined aerobic–resistance training and sustained lifestyle physical activity exert more pronounced and biologically meaningful effects than single-modality interventions. Tylutka et al. [239] observed that older adults with long-term high physical activity levels were more likely to maintain a CD4/CD8 ratio within the healthy range and exhibited higher proportions of naïve T lymphocytes alongside fewer senescence-associated T-cell subsets. This pattern suggests that cumulative exercise exposure across the lifespan, rather than short-term training alone, is critical for preserving immune repertoire diversity. Accordingly, Turner [206] proposed that the lifetime “dose” of physical activity is inversely associated with both the onset and severity of immunosenescence.

Quantitative dose–response evidence further supports this integrated view. The meta-analysis by Khalafi et al. [240], encompassing 40 studies and 1898 older adults, demonstrated that once a minimum effective training dose is reached, aerobic, resistance, and combined exercise programs all significantly reduce TNF-α and CRP, with aerobic exercise producing the largest effect sizes. This suggests the presence of a threshold effect, beyond which additional training yields diminishing returns but still contributes to immune maintenance. At the population level, Chastin et al. [241] showed that individuals in the highest physical activity quantiles had a 31% lower risk of community-acquired infections, reinforcing the concept that higher cumulative exercise doses are associated with clinically meaningful reductions in infection susceptibility.

Traditional Chinese mind–body exercises offer an additional modality well suited for achieving long-term exercise accumulation in older adults. Yang et al. [194] demonstrated that a 5-month Taiji–Qigong intervention improved influenza vaccine antibody responses, indicating preservation of immune responsiveness. The meta-analysis by Oh et al. [242] further confirmed improvements in multiple immune-related parameters following Tai Chi and Qigong practice. Although their acute physiological stimulus is modest, these exercises may contribute meaningfully to immunosenescence attenuation by enabling sustained adherence and long-term dose accumulation. Supporting evidence from local studies suggests that practices such as Baduanjin, Wu Qin Xi, and Yijinjing can increase T-lymphocyte and NK cell numbers and modulate the gut microbiota composition, potentially influencing the gut–immune axis.

Finally, temporal characteristics of exercise exposure are critical to understanding dose–response relationships. Sellami et al. [243] reported that acute exercise induces transient immune activation (e.g., increased salivary IgA), whereas chronic training over weeks to months leads to sustained reductions in inflammatory markers and adaptive immune remodeling. Taken together, these findings indicate that immunosenescence attenuation is most effectively achieved through the accumulation of a sufficient “total exercise dose” over time, rather than through sporadic or short-term interventions.

In synthesis, a regimen centered on moderate-intensity aerobic exercise, complemented by resistance training and sustained mind–body or lifestyle physical activity, appears most effective for attenuating immunosenescence. The benefits manifest at multiple levels, including the immune cell composition (e.g., preservation of naïve T cells and normalization of CD4/CD8 ratio), the inflammatory milieu (reduced TNF-α and CRP), and functional outcomes (enhanced vaccine responsiveness and lower infection risk) (Table 1 and Table 2).

## 5. Clinical Implications, Public Health Value, and Future Research Directions

The accumulated evidence reviewed above has important clinical and public health implications at multiple levels. First, with respect to infection risk, higher levels of habitual physical activity are associated with reductions in incident infections, directly counteracting the increased susceptibility to infection that characterizes immunosenescence. Second, regarding vaccine effectiveness, exercise has been shown to prolong the duration of protection conferred by influenza vaccination and to enhance antibody responses, which is particularly valuable during influenza seasons and may also inform optimization of vaccination strategies for newer vaccines, such as those against SARS-CoV-2. Third, in terms of attenuating immunosenescence, regular physical activity can normalize the CD4/CD8 ratio, increase the proportion of naïve T cells, and reduce senescence-associated T-cell subsets, thereby functionally “rejuvenating” the aging immune system. Fourth, concerning chronic low-grade inflammation, exercise consistently lowers CRP, TNF-α, and IL-6, thereby helping to prevent inflammation-related chronic diseases. Finally, from a health economics and implementation standpoint, exercise interventions are low-cost, scalable, and generally free of serious adverse effects, making them an ideal strategy for maintaining immune health in older adults, especially in resource-limited community and healthcare settings.

Notably, however, the magnitude and consistency of these beneficial effects are subject to substantial inter-individual variability, with sex differences, the comorbidity burden, and the baseline inflammatory status emerging as major determinants of responsiveness. Sex-specific trajectories of immune aging have been increasingly recognized, with older men and women exhibiting distinct patterns of immunosenescence and inflammaging. For example, males tend to show earlier contraction of naïve T-cell pools and greater expansion of terminally differentiated effector T cells, whereas postmenopausal females often exhibit higher baseline levels of systemic inflammatory markers such as CRP and IL-6 [244,245]. These biological differences—driven by sex hormones, sex chromosomes, and epigenetic regulation—may partially explain the sex-dependent variability in immune responses to exercise interventions, although most existing trials are not sufficiently powered for sex-stratified analyses [244,246].

Comorbidities further modulate exercise–immune interactions. Older adults with cardiometabolic diseases, autoimmune disorders, or neurodegenerative conditions often present with more pronounced inflammaging phenotypes and altered immune cell compositions, which may influence both the direction and magnitude of exercise-induced immune adaptations. Evidence from meta-analytic data suggests that reductions in IL-6 following exercise training are more evident in older adults with chronic diseases than in relatively healthy counterparts, indicating disease-specific dose–response patterns [247,248]. Medication use, disease severity, and multimorbidity may therefore act as important effect modifiers that are insufficiently controlled for in many intervention studies.

Baseline inflammatory status represents an additional source of variability. Individuals with elevated pre-intervention inflammatory markers may experience greater absolute reductions following exercise training, whereas those with low baseline inflammation may show attenuated or heterogeneous responses. Such “ceiling” or “floor” effects are rarely accounted for in conventional group-level analyses and may obscure responder versus non-responder phenotypes [245,247,248]. Medication use, disease severity, and multimorbidity may therefore act as important effect modifiers that are insufficiently controlled for in many intervention studies. Together, these factors highlight the need to interpret summarized outcomes within the context of participant heterogeneity rather than as uniform effects applicable to all older adults.

Despite the growing body of evidence, several important knowledge gaps remain. With regard to the optimal exercise dose, more research is needed to develop individualized exercise prescriptions for older adults with diverse health statuses (e.g., frailty, multimorbidity), particularly studies that tailor exercise type, intensity, frequency, and duration to different stages and severities of immunosenescence. In terms of long-term outcomes, relatively few intervention studies extend beyond 10 months; the cumulative effects of the lifelong exercise “dose” and the existence of critical windows for exercise across the life course (e.g., midlife vs. late life) warrant elucidation through large, prospective cohort studies. At the mechanistic level, the molecular pathways through which exercise ameliorates immunosenescence remain incompletely understood; future work should explore mechanisms involving epigenetic regulation, mitochondrial function, autophagy, and inter-organ communication networks. For traditional Chinese exercise modalities, although preliminary findings are promising, more high-quality randomized controlled trials and mechanistic studies are needed to clarify their unique pathways of action and to identify populations that may benefit most. Finally, in special populations—such as frail older adults, those with cognitive impairment, and individuals with multiple chronic conditions—there is an urgent need to develop and test tailored exercise programs and to investigate the combined effects of exercise with pharmacological, nutritional, and other lifestyle interventions, so as to construct multi-dimensional strategies for the comprehensive management of immunosenescence.

In summary, there is clear evidence for a dose–response relationship between different exercise modalities and the attenuation of immunosenescence in older adults. Moderate-intensity aerobic training performed for at least 10 months appears necessary to achieve durable effects; resistance training requires the appropriate load and frequency to effectively remodel immune cell function; traditional mind–body exercises can elicit measurable benefits within approximately 5 months and offer strong cultural acceptability; and the cumulative lifetime dose of lifestyle physical activity exerts a profound influence on the tempo of immunosenescence. Importantly, these effects are modulated by sex, comorbidities, and baseline inflammatory phenotypes, underscoring the need for precision exercise prescriptions in aging populations. Together, these findings provide a solid scientific basis for designing precise, individualized exercise prescriptions for older adults and lend strong support to the application of the “exercise is medicine” paradigm in the prevention and management of immunosenescence.

## 6. Conclusions

Immunosenescence, characterized by a progressive decline in immune function with age, leads to significant impairments in T- and B-cell responses, the reduced efficacy of dendritic cells, and diminished natural killer cell activity, ultimately decreasing the capacity to fight infections and clear tumors. This decline increases susceptibility to autoimmune diseases, chronic inflammation, and cancer, underscoring the urgent need for effective interventions. Exercise emerges as a transformative strategy to combat immunosenescence by inducing metabolic remodeling that enhances insulin sensitivity, regulates immune cell phenotypes, and reduces chronic inflammation through the mTOR and AMPK signaling pathways. Furthermore, exercise promotes an optimal balance in immune responses by modulating lactate levels and supporting the transition from pro-inflammatory to anti-inflammatory states, effectively sustaining immune function in aging individuals. Exercise-induced lipid and amino acid metabolic changes play crucial roles in improving immune function by reducing visceral fat accumulation and optimizing amino acid metabolism, leading to restored immune cell functionality and healthier immune profiles in older adults. The comprehensive organ–immune crosstalk facilitated by exercise, particularly through the release of myokines and modulation of the gut microbiota, enhances immune cell activity and contributes to systemic immune regulation, countering age-related immune decline. Notably, exercise effectively remodels both innate and adaptive immune cells by promoting the functionality of neutrophils, macrophages, and T cells while augmenting naive T-cell output from the thymus. These adaptations improve immune surveillance and response, reinforcing the assertion that exercise is vital for delaying the aging-related decline in immune health. Various exercise modalities, especially moderate-intensity aerobic and combined aerobic–resistance training, have proven effective at reducing chronic inflammation and enhancing immune responses in older adults, emphasizing the need for a sustained “dose” of physical activity to rejuvenate the immune system. This evidence underscores the importance of optimizing exercise prescriptions and individualizing interventions, which are essential for advancing public health strategies aimed at improving immune health and addressing the challenges of immunosenescence in aging populations.

## Figures and Tables

**Table 1:** Effects of different exercise modalities on immune function in older adults.

Exercise Modality	Main Immune Outcomes	Representative Immune Markers	Proposed Mechanisms
Aerobic exercise [236,237,238]	Prolonged duration of vaccine-induced protection; enhanced antibody responses to influenza vaccination; reduction in systemic low-grade inflammation	Improved influenza vaccine antibody titers and seroprotection after ~10 months of training; reductions in CRP, TNF-α, and IL-6; favorable changes in T-cell subset distribution	Moderate-intensity cardiovascular training enhances humoral responses to vaccination and downregulates chronic systemic inflammation, thereby counteracting aspects of immunosenescence
Resistance training [23,239]	Improved immune cell function; reduction in pro-inflammatory markers; potential reduction in infection susceptibility	Decreased CRP with a moderate effect size; significant reductions in IL-10 and TNF-α; trend towards lower IL-6; improvements in functional indices of immune cells	Increases muscle mass and improves metabolic status, which, in turn, modulate inflammatory cytokines and immune cell function, contributing to attenuation of age-related chronic inflammation
Combined aerobic and resistance training/high lifestyle physical activity [206,240]	Attenuation of immunosenescence-related phenotypes; optimization of CD4/CD8 ratio; improvement in naïve vs. senescent T-cell balance	Higher prevalence of CD4/CD8 ratio within the healthy range in active vs. sedentary older adults; increased proportion of naïve T cells; reduced frequencies of senescence-associated T-cell subsets	Long-term, higher-volume lifestyle physical activity promotes “immune rejuvenation” by maintaining a more youthful T-cell profile and mitigating age-related immune remodeling
Tai Chi and Qigong [194,243]	Improved vaccine responses; enhancement of multiple immune parameters; potential modulation of immunosenescence	Increased influenza vaccine antibody responses after a 5-month moderate-intensity Taiji–Qigong program in older adults; improvements across several immune indices in systematic reviews	Mind–body exercise combining moderate-intensity physical activity, breathing regulation, and relaxation exerts systemic effects on neuroendocrine–immune interactions, enhancing vaccine responsiveness and overall immune regulation

**Table 2:** Effects of exercise dosage parameters on immune function in older adults and specific immune markers.

Exercise Dosage Parameter	Recommended Protocol	Improvements in Specific Immune Markers	Effects on Immune Function	Dose–Response Relationship
Exercise intensity				
Moderate intensity [236,237,240]	• Most commonly recommended • Suitable for the majority of older adults • Highest safety profile	Humoral immunity: • Increased mean fold rise in influenza vaccine antibodies • Ten-month training prolongs antibody protection Cellular immunity: • Optimized CD4/CD8 ratio • >50% of physically active individuals with ratios within the healthy range Cytokines: • TNF-α and CRP significantly reduced • IL-6 reduced in older adults with chronic diseases	• Ten months of moderate-intensity aerobic exercise improves antibody responses • Moderate intensity is more appropriate than high intensity for older adults • Marked effect in attenuating immunosenescence	Moderate intensity is positively associated with improvements in immune function; multiple RCTs and meta-analyses support it as the optimal intensity
Resistance training intensity [23,239]	• Tailored to individual status • Tolerance should be evaluated • Professional supervision recommended	Cytokines: • CRP reduced with a moderate effect size • Meta-analysis of 18 RCTs shows significant reductions in TNF-α and IL-10 • IL-6 shows a downward trend Cellular immunity: • Improved immune cell function • Reduced infection risk	• Systematic review confirms broad benefits for immune cell function • Decreases susceptibility to infection • Improves vaccine effectiveness	Dose of resistance training is inversely associated with inflammatory markers, indicating a dose–response relationship
Exercise frequency and duration				
10-month long-term training [236,237]	• Cardiovascular exercise • Moderate intensity • Regular weekly training	Humoral immunity: • Prolonged seroprotection following influenza vaccination • Significantly improved antibody responses • Protective effect maintained for 10 months Systemic effects: • Marked immune improvements in sedentary older adults	• RCTs confirm that 10-month training extends vaccine protection • Particularly effective in previously sedentary older adults • Continuous, regular exercise is key	Long-term training (10 months) yields more sustained immune protection than short-term interventions
5-month Taiji–Qigong program [194]	• Several sessions per week • Moderate-intensity practice • Traditional exercise modality	Humoral immunity: • Improved influenza vaccine antibody responses • Significantly enhanced immune reaction	• As little as 5 months of practice can improve vaccine responses • Gentle modality well suited to older adults • High safety and adherence	Five months of moderate-intensity Taiji–Qigong leads to significant improvements in vaccine responses
Combined effects of different exercise modalities [241,242]	• Aerobic, resistance, and combined training • Individualized selection • Long-term adherence	Inflammatory markers (meta-analysis): • TNF-α and CRP significantly reduced • IL-6 significantly reduced in older adults with chronic diseases • Magnitude of TNF-α reduction: aerobic > combined > resistance • Small-to-moderate effect sizes	• All exercise modalities are beneficial for inflammatory markers • Aerobic exercise shows the strongest effect on TNF-α • Older adults with chronic diseases derive greater benefits • Approximately 31% reduction in infection risk	Exercise training shows a dose–response relationship with improvements in inflammatory markers; high levels of physical activity are associated with a 31% reduction in infection risk
Lifestyle physical activity (long-term, regular) [206,240]	• Lifetime “dose” of exercise • Regular physical activity • Gradual, progressive approach	Immunosenescence: • Normalization of CD4/CD8 ratio • Increased percentage of naïve T lymphocytes • Reduced senescence-associated T cells Telomere length: • Longer telomeres in highly active individuals	• Attenuates immunosenescence and “rejuvenates” the immune system • May delay onset or reduce severity of immunosenescence • Lifetime exercise “dose” has long-term impact	Lifetime exercise “dose” is negatively associated with the degree of immunosenescence; lifestyle physical activity may delay the onset of immunosenescence or lessen its severity
Acute vs. chronic exercise [244]	• Distinguish short-term effects • Emphasize long-term adaptations • Understand systemic responses	Immunoglobulins and cytokines: • Acute endurance exercise: increased secretory salivary IgA • Short-term chronic training: reduced systemic inflammatory markers • Changes in immunoglobulin and cytokine levels • Adaptive responses of the aging immune system	• Acute exercise induces transient immune activation • Chronic regular exercise produces lasting immune improvements • The aging immune system exhibits distinct responses to exercise	Acute exercise elicits immediate effects, whereas chronic regular exercise induces long-term immune adaptation and improvement
Physical activity level and infection risk [242]	• High, regular activity levels • Maintain over time • Emphasis on prevention	Epidemiological evidence: • Individuals with high physical activity levels • 31% reduction in risk of community-acquired infections (HR = 0.69, 95% CI [0.61–0.78])	• Regular physical activity significantly reduces risk of infectious diseases • Prevents community-acquired infections • Major public health implications	Physical activity level is inversely associated with infection risk in a dose–response manner

## Data Availability

No new data were created or analyzed in this study. Data sharing is not applicable.
